# Preoperative serum HE4 combined with CA125 testing for predicting the risk of suboptimal cytoreduction in advanced epithelial ovarian cancer

**DOI:** 10.3389/fonc.2026.1886007

**Published:** 2026-09-01

**Authors:** Chun Zhou, Yanli Wu, Juan Wang, Fu Zheng

**Affiliations:** 1 Department of Obstetrics and Gynecology, Union Jiangnan Hospital (The First People’s Hospital of Jiangxia District), Wuhan, Hubei, China; 2 Department of Oncology, The First People’s Hospital of Guangshui, Guangshui, Hubei, China; 3 Chongqing Preschool Education College, Chongqing, China; 4 Department of Obstetrics and Gynecology, Gezhouba Central Hospital of Sinopharm/The Third Clinical Medical College, China Three Gorges University, Yichang, Hubei, China

**Keywords:** carbohydrate antigen 125, cytoreductive surgery, human epididymis protein 4, ovarian cancer, platelet count, prediction model

## Abstract

**Objective:**

To investigate the predictive value of preoperative serum markers centered on human epididymis protein 4 (HE4) combined with carbohydrate antigen 125 (CA125) for the risk of suboptimal cytoreduction in advanced epithelial ovarian cancer.

**Methods:**

We retrospectively analyzed the clinical data of 102 patients with International Federation of Gynecology and Obstetrics (FIGO) stage IIIA–IV epithelial ovarian cancer who underwent primary cytoreductive surgery at the First People’s Hospital of Jiangxia District between January 2021 and December 2025. Patients were divided into an optimal cytoreduction group (n=65) and a suboptimal cytoreduction group (n=37) based on the size of postoperative residual lesions. Preoperative HE4 and CA125 levels were compared between the two groups. Restricted cubic spline (RCS) functions were used to analyze dose–response relationships. Multivariate Logistic regression was applied to screen independent risk factors and construct a combined prediction model. Model performance was evaluated using receiver operating characteristic (ROC) curves, calibration curves, and decision curve analysis (DCA).

**Results:**

Preoperative HE4, CA125, and platelet counts were significantly higher in the suboptimal cytoreduction group than in the optimal cytoreduction group (all P<0.001). RCS analysis showed a linear positive correlation between marker levels and the risk of suboptimal cytoreduction. Multivariate regression revealed that each 50 pmol/L increase in HE4 (odds ratio [OR]=1.25), each 100 U/mL increase in CA125 (OR = 1.68), and each 10×10^9^/L increase in platelets (OR = 1.27) were independent risk factors (all P<0.05). The combined model achieved an area under the ROC curve (AUC) of 0.959 for predicting suboptimal cytoreduction, with a sensitivity of 81.1%, specificity of 95.4%, and accuracy of 90.2%, significantly outperforming single markers. Calibration curves showed good model fit (P = 0.431), and DCA demonstrated significant net clinical benefit at threshold probabilities ranging from 0.1 to 0.9.

**Conclusion:**

Combined preoperative detection of serum HE4, CA125, and platelet count shows preliminary predictive potential for the risk of suboptimal cytoreduction in advanced epithelial ovarian cancer. In this single-center retrospective cohort, the three-marker combined model demonstrated promising internal predictive performance; however, its clinical applicability remains preliminary and center-specific, pending validation in independent external cohorts.

## 1 Introduction

Ovarian cancer is the most lethal malignant tumor of the female reproductive system (1). According to epidemiological estimates, there were approximately 290,000 new cases of ovarian cancer worldwide annually in 2021, and this number is projected to rise to 470,000 by 2050 (2, 3). Because the ovaries are located deep in the pelvic cavity, early lesions lack specific symptoms. More than 70% of patients are diagnosed at an advanced stage, with a 5-year overall survival rate of less than 40% (4). Cytoreductive surgery is the core treatment for ovarian cancer. The size of postoperative residual lesions is an independent prognostic factor, and achieving gross total resection (R0 resection) is the primary surgical goal (5). However, advanced ovarian cancer is often accompanied by extensive peritoneal implantation and multiorgan involvement. Accurate preoperative assessment of tumor burden, surgical difficulty, and required resection scope remains a clinical challenge in gynecologic oncology (6, 7). Insufficient surgical resection leads to residual tumor and compromises patient survival. Overly extensive resection increases the risk of intraoperative injury, postoperative complications, and perioperative mortality, severely impairing postoperative quality of life.

Current preoperative evaluation of ovarian cancer mainly relies on imaging examinations such as enhanced pelvic and abdominal computed tomography (CT)/magnetic resonance imaging (MRI) and positron emission tomography–computed tomography (PET–CT) (8, 9). Nevertheless, imaging has limited sensitivity in detecting tiny peritoneal implants smaller than 5 mm and occult mesenteric metastases. Evaluation results are also highly affected by the experience of radiologists, leading to clear limitations. Serum tumor marker testing is simple, reproducible, and objective (10). Carbohydrate antigen 125 (CA125) is the most widely used marker in ovarian cancer diagnosis and management, but its specificity is relatively low. It can be elevated in benign conditions including endometriosis, pelvic inflammatory disease, and menstruation, resulting in limited value for predicting surgical risk when used alone (11). Human epididymis protein 4 (HE4) is a newly identified ovarian cancer–specific marker. It is rarely expressed in benign gynecologic diseases and is unaffected by menopausal status or menstrual cycle. HE4 is closely related to ovarian cancer invasiveness and tumor burden (12). Most existing studies focus on the diagnostic value of HE4 combined with CA125 for ovarian cancer (13). The guiding value of HE4 plus CA125 for surgical resection scope, especially preoperative assessment of optimal cytoreduction, remains scarce in domestic literature.

This retrospective study focuses on preoperative risk stratification for cytoreductive surgery, a key surgical clinical issue. We systematically explore the predictive value of preoperative serum HE4, CA125, and platelet count for suboptimal cytoreduction. The results are expected to provide preliminary evidence for establishing a simple and objective preoperative surgical risk stratification tool. This tool can help predict surgical difficulty preoperatively and assist clinicians in designing individualized resection plans. It may also support rational selection of initial treatment strategies, thereby improving the rate of optimal cytoreduction and surgical outcomes in advanced ovarian cancer.

## 2 Materials and methods

### 2.1 Study subjects

This was a single-center retrospective analysis. Consecutive patients with advanced epithelial ovarian cancer who underwent primary cytoreductive surgery at the Gynecologic Oncology Center of the First People’s Hospital of Jiangxia District between January 2021 and December 2025 were enrolled. The study was approved by the Ethics Committee of the First People’s Hospital of Jiangxia District (Ethics Approval No.: 2026-K012) and complied with the ethical requirements of the Declaration of Helsinki. Written informed consent from the patients/participants was not required to participate in this study in accordance with the national legislation and the institutional requirements.

### 2.2 Inclusion and exclusion criteria

Inclusion criteria: ① Postoperative paraffin pathology confirmed primary epithelial ovarian cancer; ② International Federation of Gynecology and Obstetrics (FIGO) stage IIIA–IVB; ③ Serum HE4 and CA125 tests completed at the First People’s Hospital of Jiangxia District within 1 week before surgery, with complete and traceable clinical data; ④ Primary cytoreductive surgery performed at the First People’s Hospital of Jiangxia District, with complete surgical records and pathological reports. Exclusion criteria: ① Received any antitumor treatment before surgery, including neoadjuvant chemotherapy, radiotherapy, targeted therapy, or endocrine therapy; ② Complicated with other primary malignant tumors; ③ Complicated with acute or chronic infection, autoimmune disease, severe hepatic or renal insufficiency, heart failure, hematological disease, or other conditions that may affect serum HE4, CA125 levels, or platelet count; ④ Non-epithelial ovarian malignant tumors such as borderline ovarian tumor, germ cell tumor, or sex cord–stromal tumor; ⑤ Missing clinicopathological data that prevented statistical analysis. Finally, a total of 102 eligible patients were included. Of note, intraoperative surgical complexity metrics such as the Peritoneal Cancer Index (PCI) or the Aletti Surgical Complexity Score were not systematically recorded in our institutional database, and therefore could not be incorporated into the present analysis.

### 2.3 Surgical protocol and outcome definition

All surgeries were performed by senior attending physicians or above in the Gynecologic Oncology Group of the First People’s Hospital of Jiangxia District (with ≥15 years of experience in gynecologic oncology surgery). Surgical protocols strictly followed the guidelines for the diagnosis and treatment of ovarian malignant tumors, with the core goal of achieving gross total resection (14). The standard surgical scope included total hysterectomy + bilateral salpingo-oophorectomy + omentectomy ± pelvic lymphadenectomy. Extended cytoreductive surgery was performed for patients with preoperative or intraoperative evidence of upper abdominal involvement, paraaortic lymph node metastasis, or adjacent organ invasion. Outcomes were determined based on surgical records and immediate postoperative assessment by the primary surgeon. Optimal cytoreduction group (Y = 0) was defined as no gross residual lesions in the abdominal cavity or single residual lesion with maximum diameter < 1 cm at the end of surgery. Suboptimal cytoreduction group (Y = 1) was defined as residual lesion with maximum diameter ≥ 1 cm. This binary outcome served as the dependent variable in the Logistic regression model.

### 2.4 Data collection and quality control

All clinical data were independently extracted from the electronic medical record system, laboratory system, surgical records, and pathological reports of the First People’s Hospital of Jiangxia District by two uniformly trained gynecologic oncology specialists. Cross-checking was performed after extraction. Discrepant data were adjudicated by a third chief physician of gynecologic oncology. Collected data included: ① General data: age, menopausal status; ② Clinicopathological data: FIGO stage, pathological type; ③ Preoperative laboratory data: serum HE4, CA125 levels, and platelet count within 1 week before surgery; ④ Surgery-related data: surgical outcome.

### 2.5 Statistical analysis

**Figure 1:**
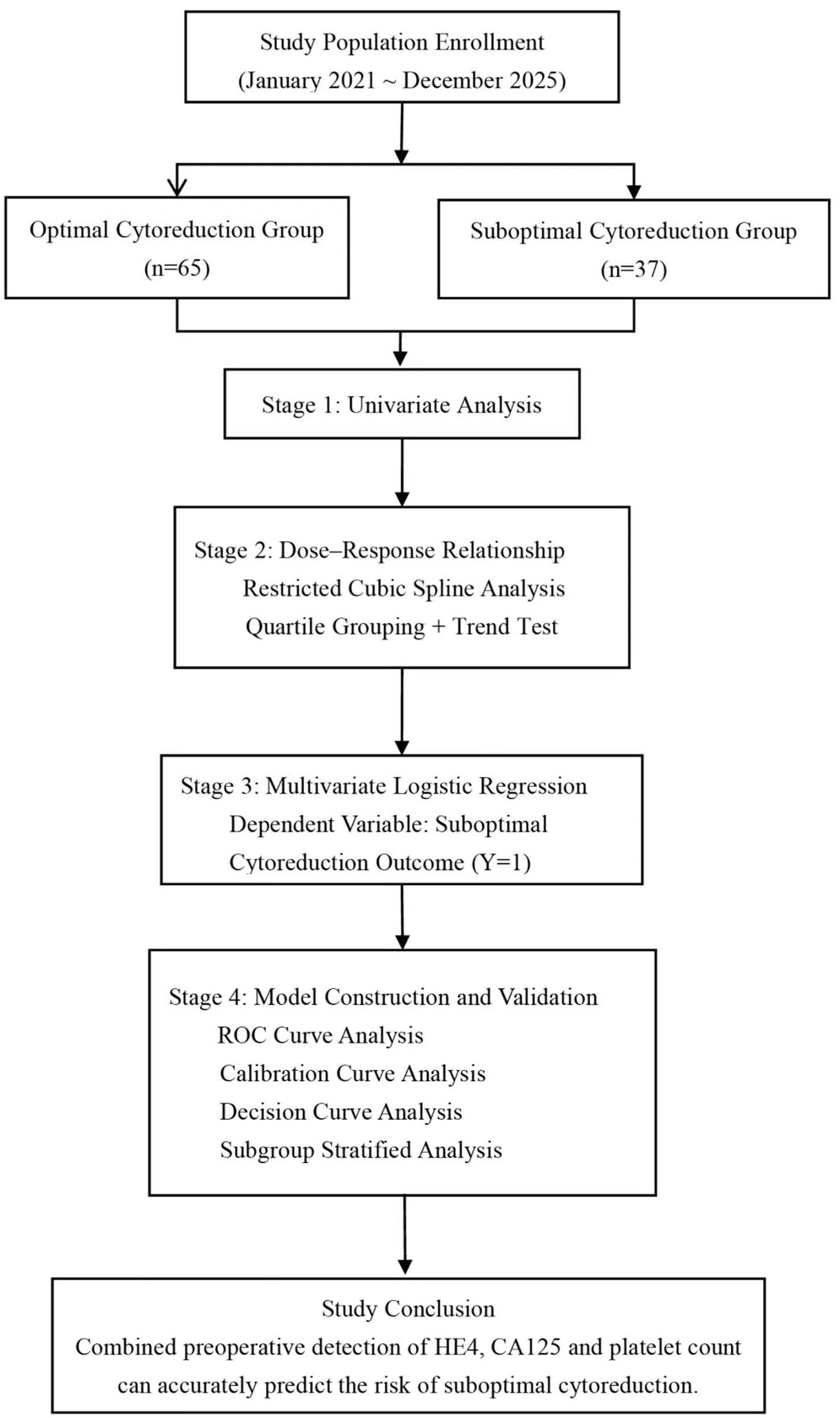
Flowchart of the study.

Statistical analyses were performed using SPSS 26.0 and R 4.3.1 software. This was a predictive model study. The core dependent variable Y was suboptimal cytoreduction: Y = 1 for suboptimal cytoreduction and Y = 0 for optimal cytoreduction. The core independent variables were preoperative serum HE4, CA125 levels, and platelet count. The study flowchart is shown in **Figure 1**. Measurement data with normal distribution were presented as mean ± standard deviation, and between-group comparisons were performed using independent-samples t-test. Non-normally distributed measurement data were presented as median and interquartile range M (Q1, Q3), and between-group comparisons were performed using Mann-Whitney U test. Enumeration data were presented as case number and percentage n (%), and between-group comparisons were performed using χ^2^ test or Fisher exact test. Receiver operating characteristic (ROC) curves were used to evaluate the predictive performance of HE4, CA125, platelet count, and the three-marker combined model for suboptimal cytoreduction. The area under the curve (AUC), sensitivity, specificity, positive predictive value (PPV), negative predictive value (NPV), and accuracy at the optimal cutoff value were calculated. AUC comparisons were performed using DeLong test. Restricted cubic spline (RCS) functions were used to analyze the nonlinear association between HE4, CA125, and the risk of suboptimal cytoreduction. Knots were placed at the 25th, 50th, and 75th percentiles, and nonlinearity was tested using the likelihood ratio test. HE4 and CA125 levels were transformed into categorical variables by quartiles, and Cochran–Armitage trend test was used to analyze the trend in the rate of suboptimal cytoreduction. To correct for sparse-data bias in the quartile-stratified logistic regression, Firth’s penalized maximum likelihood estimation was applied to calculate adjusted odds ratios with corresponding 95% confidence intervals. Variables with P<0.1 in univariate analysis and clinically important variables were entered into multivariate Logistic regression analysis. The enter method was used to screen independent risk factors, and odds ratios (OR) and 95% confidence intervals (CI) were calculated. For clinical interpretability, continuous variables were scaled by unit before being included in the model. Internal validation of the combined model was performed using the bootstrap resampling method. Calibration curves were plotted, and model goodness of fit was evaluated using the Hosmer–Lemeshow test. Decision curve analysis (DCA) was used to assess the net clinical benefit of the model at different threshold probabilities. Subgroup analysis and interaction testing were performed to evaluate the robustness of the combined model in populations with different clinical characteristics. Interaction P values were calculated using the bootstrap method. A two-sided P<0.05 was considered statistically significant.

## 3 Results

### 3.1 Comparison of baseline characteristics and serum marker levels between the two groups

A total of 102 patients with primary epithelial ovarian cancer were enrolled in this study, including 65 cases (63.7%) in the optimal cytoreduction group and 37 cases (36.3%) in the suboptimal cytoreduction group. There were no significant differences in age, menopausal status, FIGO stage distribution, or pathological type between the two groups (all P>0.05) (**Table 1**). Serum marker detection showed that the median preoperative HE4 level in the optimal cytoreduction group was 225.0 pmol/L, which was significantly lower than 472.8 pmol/L in the suboptimal cytoreduction group, with a statistically significant difference (P<0.001). The median preoperative CA125 level in the optimal cytoreduction group was 304.7 U/mL, which was significantly lower than 871.3 U/mL in the suboptimal cytoreduction group (P<0.001). In addition, the preoperative platelet count in the optimal cytoreduction group was (253.3 ± 89.6)×10^9^/L, which was significantly lower than (332.7 ± 62.1)×10^9^/L in the suboptimal cytoreduction group.

**Table 1:** Comparison of baseline characteristics and serum marker levels between the two groups of ovarian cancer patients.

Variable	Overall (n=102)	Optimal cytoreduction group (n=65)	Suboptimal cytoreduction group (n=37)	P-value
Age (years)	56.1 ± 9.4	56.2 ± 9.9	56.0 ± 8.7	0.935
Menopausal status [n(%)]				1.000
Premenopausal	43 (42.2)	27 (41.5)	16 (43.2)	
Postmenopausal	59 (57.8)	38 (58.5)	21 (56.8)	
FIGO stage [n(%)]				0.102
Stage III	72 (70.6)	50 (76.9)	22 (59.5)	
Stage IV	30 (29.4)	15 (23.1)	15 (40.5)	
Pathological type [n(%)]				0.075
High-grade serous carcinoma	87 (85.3)	59 (90.8)	28 (75.7)	
Other types	15 (14.7)	6 (9.2)	9 (24.3)	
Preoperative HE4 (pmol/L)	271.6 (190.9-471.6)	225.0 (152.2-315.7)	472.8 (325.8-548.0)	<0.001
Preoperative CA125 (U/mL)	442.4 (265.4-837.2)	304.7 (206.6-459.4)	871.3 (673.7-1344.2)	<0.001
Platelet count (×10^9^/L)	282.1 ± 89.1	253.3 ± 89.6	332.7 ± 62.1	<0.001

### 3.2 Nonlinear relationship analysis of preoperative HE4 and CA125 levels with the risk of suboptimal cytoreduction

**Figure 2:**
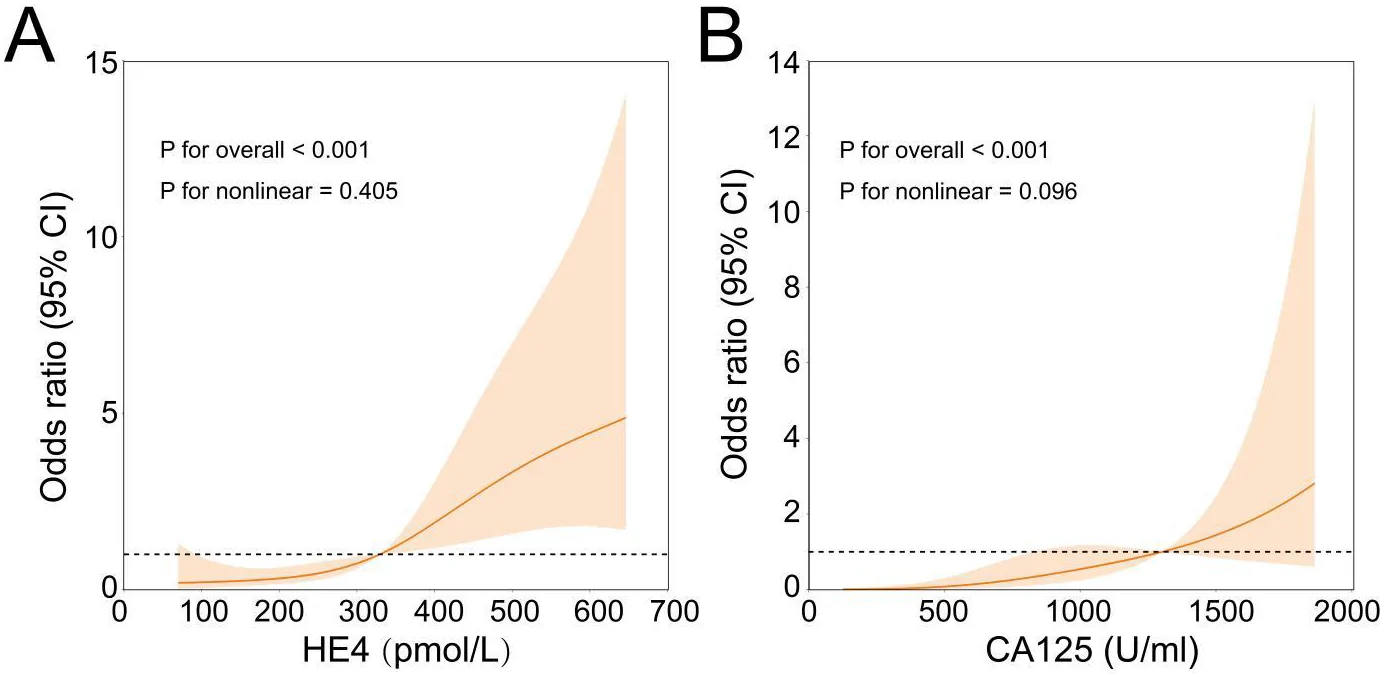
Restricted cubic spline analysis of preoperative serum marker levels. **(A)** RCS curve of preoperative HE4 level and the risk of suboptimal cytoreduction; **(B)** RCS curve of preoperative CA125 level and the risk of suboptimal cytoreduction.

To clarify the association pattern between serum marker levels and surgical outcomes, RCS functions were used to analyze the dose–response relationship of HE4 and CA125 with the risk of suboptimal cytoreduction, respectively. RCS analysis results showed that there was a significant overall association between preoperative HE4 level and the risk of suboptimal cytoreduction (P_overall_<0.001), but the curve shape showed no obvious nonlinear characteristics, and the nonlinear test result was P_nonlinear_=0.405 (**Figure 2A**). Similarly, the overall association between preoperative CA125 level and the risk of suboptimal cytoreduction was statistically significant (P_overall_<0.001), and its risk curve showed a monotonically increasing trend with increasing values, while the nonlinear test result was not statistically significant (P_nonlinear_=0.096) (**Figure 2B**). The above results indicated that elevated HE4 and CA125 levels were both approximately linearly and positively correlated with an increased risk of suboptimal cytoreduction, supporting the use of linear assumptions for subsequent model construction.

### 3.3 Dose–response relationship and trend test of preoperative HE4 and CA125 levels for the risk of suboptimal cytoreduction

This section presents a partially adjusted model (adjusted for age, FIGO stage, and platelet count) designed primarily to quantify the dose–response relationship and trend across quartiles of HE4 and CA125 levels, rather than to identify independent predictors. The fully adjusted multivariable model identifying independent risk factors is presented separately in Section 3.4. Given the linear trend suggested by RCS analysis, patients were further grouped according to quartiles of HE4 and CA125 levels to quantify risk differences in different level intervals and test the dose–response trend (**Table 2**). With HE4 level increasing from Q1 group to Q4 group, the incidence of suboptimal cytoreduction was 11.5%, 12.0%, 48.0%, and 73.1% in turn, and Cochran–Armitage trend test showed that the upward trend was statistically significant (P~trend~ < 0.001). After applying Firth’s penalized maximum likelihood logistic regression to correct for sparse-data bias, with adjustment for age, FIGO stage, and platelet count, compared with Q1 group, the risk of suboptimal cytoreduction was significantly increased in Q3 and Q4 groups for HE4, with adjusted OR values of 5.92 (95%CI: 1.46–29.69, P = 0.012) and 16.86 (95%CI: 3.89–97.75, P < 0.001), respectively. A similar pattern was observed for CA125 level, with adjusted OR values of 17.18 (95%CI: 2.75–203.21, P = 0.001) for Q3 and 272.37 (95%CI: 29.61–5549.32, P < 0.001) for Q4. Notably, although the precision of the CA125 Q4 estimate was limited due to the near-complete separation and small sample size in this subgroup, the highly significant trend (P~trend~ < 0.001) robustly confirmed the dose–response relationship. Unit increment analysis showed that for every 50 pmol/L increase in HE4, the risk of suboptimal cytoreduction increased by 35% (adjusted OR = 1.35, 95%CI: 1.17–1.60, P < 0.001). For every 100 U/mL increase in CA125, the risk increased by 74% (adjusted OR = 1.74, 95%CI: 1.41–2.34, P < 0.001).

**Table 2:** Dose-response relationship between preoperative HE4, CA125 levels and the risk of suboptimal debulking.

Variable	Range	Cases	Proportion of suboptimal cytoreduction	Adjusted OR (95% CI)^a^	P-value	P for trend^b^
HE4 quartile groups						<0.001
Q1	<190.9 pmol/L	26	3 (11.5)	Reference		
Q2	190.9~271.6 pmol/L	25	3 (12.0)	1.64 (0.28–9.87)	0.574	
Q3	271.6~471.6 pmol/L	25	12 (48.0)	5.92 (1.46–29.69)	0.012	
Q4	≥471.6 pmol/L	26	19 (73.1)	16.86 (3.89–97.75)	<0.001	
CA125 quartile groups						<0.001
Q1	<265.4 U/mL	26	1 (3.8)	Reference		
Q2	265.4~442.4 U/mL	25	4 (16.0)	8.47 (1.01–117.7)	0.049	
Q3	442.4~837.2 U/mL	25	11 (44.0)	17.18 (2.75–203.21)	0.001	
Q4	≥837.2 U/mL	26	21 (80.8)	272.37 (29.61–5549.32)	<0.001	
Unit increment analysis						
Per 50 pmol/L increase in HE4	—	—	—	1.35 (1.17-1.60)	<0.001	
Per 100 U/mL increase in CA125	—	—	—	1.74 (1.41-2.34)	<0.001	

OR values were calculated using Firth’s penalized maximum likelihood logistic regression with adjustment for age, FIGO stage, and platelet count. P for trend was derived from the Cochran-Armitage trend test. Due to the limited sample size and near-complete separation in the high CA125 subgroup, the precision of the CA125 Q4 estimate is limited; however, the monotonic upward trend remains unequivocally significant.

^a^ Firth’s penalized maximum likelihood logistic regression.

^b^Cochran-Armitage trend test.

### 3.4 Multivariate logistic regression analysis of risk factors for suboptimal cytoreduction

This section presents the final fully adjusted multivariable Logistic regression model, which was constructed to identify independent risk factors for suboptimal cytoreduction after controlling for all clinically relevant covariates. Variables with statistically significant differences in univariate analysis and clinically concerned variables were included in the multivariate Logistic regression model. For clinical interpretability, continuous variables were scaled by unit before inclusion in the analysis. The results showed that after adjusting for age and FIGO stage, elevated HE4 level, elevated CA125 level, and elevated platelet count were all independent risk factors for suboptimal cytoreduction. Specifically, for every 50 pmol/L increase in HE4, the risk of suboptimal cytoreduction increased by 25% (OR = 1.25, 95%CI: 1.02–1.53, P = 0.030). For every 100 U/mL increase in CA125, the risk increased by 68% (OR = 1.68, 95%CI: 1.29–2.19, P<0.001). For every 10×10^9^/L increase in platelet count, the risk increased by 27% (OR = 1.27, 95%CI: 1.12–1.46, P<0.001). Age had no significant effect (P = 0.548). Patients with FIGO stage IV had an upward trend in the risk of suboptimal cytoreduction compared with stage III patients, but the difference was not statistically significant (OR = 4.64, 95%CI: 0.89–24.07, P = 0.068) (**Table 3**).

**Table 3:** Multivariate Logistic regression analysis of risk factors for suboptimal debulking.

Variable	β value	SE	Wald χ^2^	P-value	OR (95% CI)
HE4 (per 50 pmol/L increase)*	0.22	0.10	4.69	0.030	1.25 (1.02-1.53)
CA125 (per 100 U/mL increase)*	0.52	0.13	15.12	<0.001	1.68 (1.29-2.19)
Platelet count (per 10×10^9^/L increase)*	0.24	0.07	12.44	<0.001	1.27 (1.12-1.46)
Age	0.02	0.04	0.36	0.548	1.02 (0.95-1.10)
FIGO stage IV	1.53	0.84	3.34	0.068	4.64 (0.89-24.07)

*For better clinical interpretability, continuous variables were scaled by unit before being included in the model. The model was adjusted for age and FIGO stage.

### 3.5 Construction and diagnostic performance evaluation of the combined prediction model

**Figure 3:**
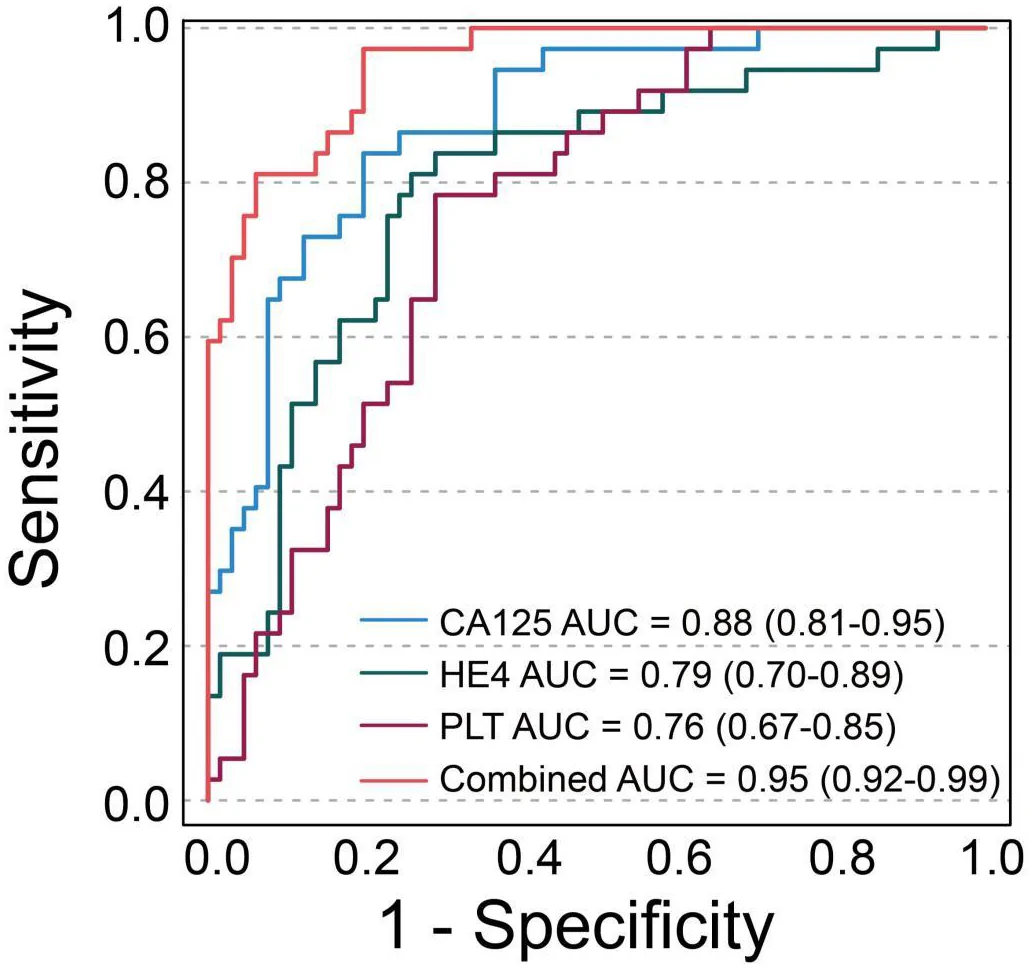
Receiver operating characteristic curves of preoperative HE4, CA125, platelet count and the combined model for predicting suboptimal cytoreduction.

Based on the results of multivariate Logistic regression analysis, a combined prediction model including HE4, CA125, and platelet count was constructed. ROC curve analysis showed that the AUC of the combined model for predicting suboptimal cytoreduction was 0.959 (95%CI: 0.926–0.992), which was significantly higher than 0.879 (95%CI: 0.812–0.946) of CA125 single index (DeLong test P = 0.006) and 0.794 (95%CI: 0.700–0.887) of HE4 single index (DeLong test P<0.001) (**Table 4**, **Figure 3**). The optimal cutoff value of the combined model was determined by the maximum Youden index, and its sensitivity for predicting suboptimal cytoreduction was 81.1%, specificity 95.4%, positive predictive value 90.9%, negative predictive value 89.7%, and overall accuracy 90.2%. All diagnostic performance indicators of the combined model were superior to single markers, especially with a significant improvement in specificity.

**Table 4:** Comparison of diagnostic performance between the combined prediction model and single markers.

Model	AUC (95% CI)	Sensitivity (%)	Specificity (%)	PPV (%)	NPV (%)	Accuracy (%)	P-value*
Combined model (HE4+CA125+PLT)	0.95 (0.92-0.99)	81.1	95.4	90.9	89.9	90.2	—
HE4 alone	0.79 (0.70-0.89)	81.1	73.8	63.8	87.3	76.5	<0.001
CA125 alone	0.88 (0.81-0.95)	83.8	80.0	70.5	89.7	81.4	0.006

PPV, positive predictive value; NPV, negative predictive value; *DeLong test was used to compare the AUC of each single marker with that of the combined model.

### 3.6 Calibration and clinical applicability assessment of the combined prediction model

**Figure 4:**
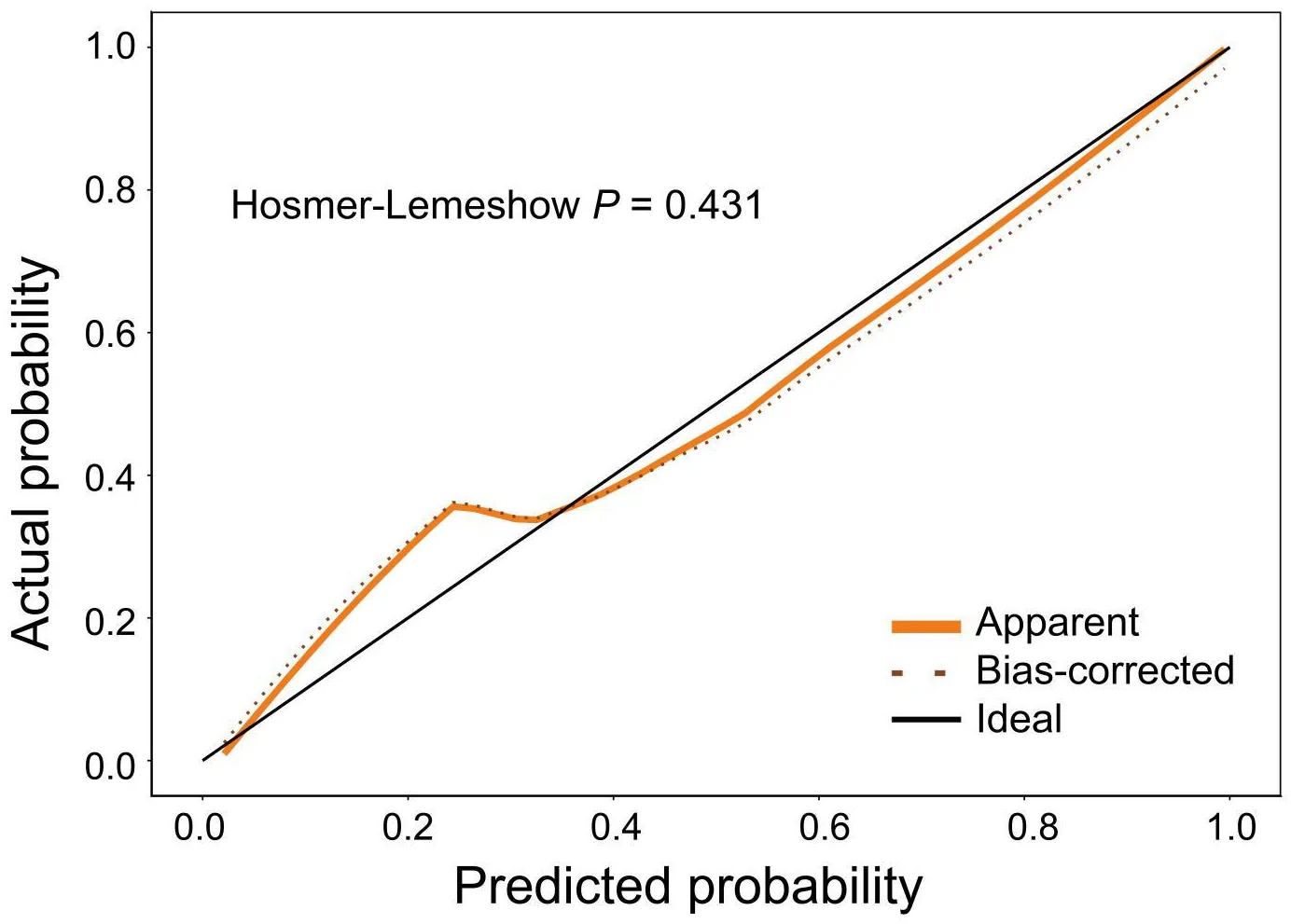
Calibration curve of the combined prediction model.

**Figure 5:**
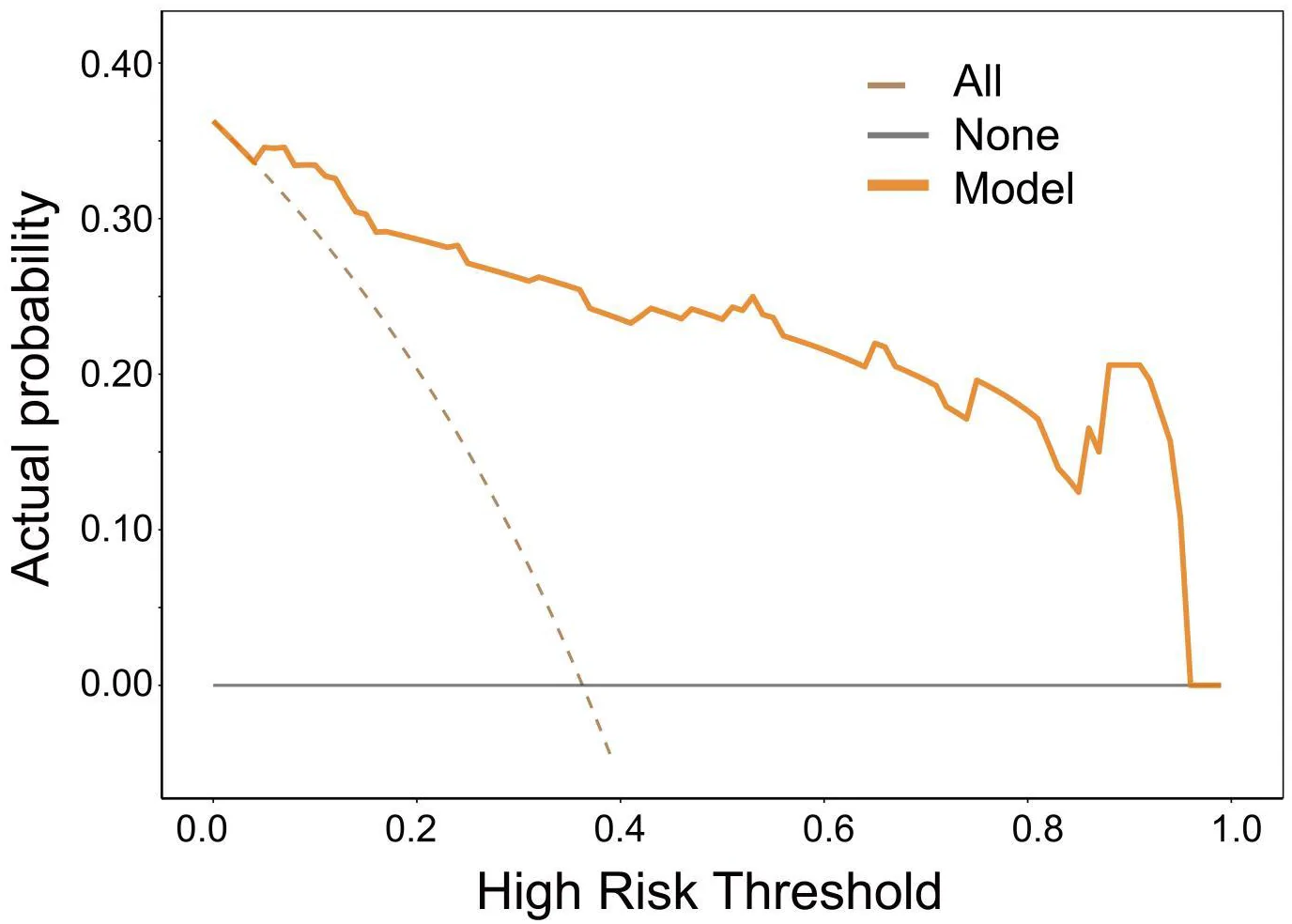
Decision curve analysis of the combined prediction model.

The bootstrap resampling method was used for internal validation of the combined model and drawing calibration curves. As shown in **Figure 4**, the predicted probability of suboptimal cytoreduction by the model was closely distributed along the 45° ideal diagonal with the actual observed probability, showing good goodness of fit. Hosmer–Lemeshow test showed no statistically significant difference (P = 0.431), indicating good model calibration. Further clinical DCA was used to evaluate the net clinical benefit of the model, and the results showed that when the risk threshold of suboptimal cytoreduction was set in a wide range of 0.1 to 0.9, the net benefit obtained by using the combined model to guide clinical decisions was higher than the extreme strategies of “all patients treated as suboptimal cytoreduction” and “all patients treated as optimal cytoreduction” (**Figure 5**). This indicated that the model had practical application value in most clinical decision-making scenarios.

### 3.7 Stratified analysis of the combined prediction model in different clinical subgroups

To evaluate the robustness and applicability of the combined model in populations with different characteristics, preset stratified analysis was performed according to menopausal status, FIGO stage, and pathological type. The results showed that the AUC of the combined model was 0.975 (95%CI: 0.939–1.000) in premenopausal patients and 0.939 (95%CI: 0.883–0.995) in postmenopausal patients, with a statistically significant difference between subgroups (P_interaction_=0.042), suggesting that the model might have higher prediction accuracy in premenopausal women. In different FIGO stage subgroups, the model maintained excellent and stable predictive performance in both stage III and IV patients, with AUC of 0.946 (95%CI: 0.898–0.995) and 0.982 (95%CI: 0.949–1.000), respectively, and no statistically significant difference between subgroups (P_interaction_=0.674). In the high-grade serous carcinoma subgroup, the model AUC was 0.942 (95%CI: 0.898–0.987); due to the small sample size of other pathological types (n=15), the AUC was not reported separately, but no significant difference was found in the subgroup interaction test (P_interaction_=0.158). The above results indicated that the combined prediction model had good universality and stability in most clinical subgroups (**Table 5**).

**Table 5:** Stratified analysis of the combined prediction model in different clinical subgroups.

Subgroup	Cases	AUC of combined model (95% CI)	P for interaction
Menopausal status			0.042
Premenopausal	43	0.975 (0.939-1.000)	
Postmenopausal	59	0.939 (0.883-0.995)	
FIGO stage			0.674
Stage III	72	0.946 (0.898-0.995)	
Stage IV	30	0.982 (0.949-1.000)	
Pathological type			0.158
High-grade serous carcinoma	87	0.942 (0.898-0.987)	
Other types*	15	—	

*Due to the small sample size of other pathological types (n=15), the AUC estimation was unstable and not reported. AUC, area under the curve; CI, confidence interval.

## 4 Discussion

This study retrospectively analyzed the data of 102 patients with advanced epithelial ovarian cancer to investigate the predictive value of preoperative serum HE4, CA125 combined with platelet count for the resection extent and optimal cytoreduction probability of cytoreductive surgery. The results showed that HE4, CA125 and platelet levels were significantly lower in the optimal cytoreduction group than in the suboptimal cytoreduction group; elevated HE4 and CA125 were linearly and positively correlated with the risk of suboptimal cytoreduction in a dose-dependent manner. Multivariate Logistic regression confirmed that elevated levels of all three indicators were independent risk factors. The combined model constructed by the three indicators achieved an AUC of 0.959, a sensitivity of 81.1%, a specificity of 95.4% and an accuracy of 90.2%, with favorable calibration and net clinical benefit, and stable predictive performance in most subgroups. These findings suggest that preoperative HE4 combined with CA125 may reflect tumor burden and invasiveness, and the addition of platelet count appears to improve the prediction of suboptimal cytoreduction risk in this single-center cohort. However, these results are exploratory and should not be interpreted as providing a definitive objective basis for individualized surgical decision-making until external validation is completed.

Preoperative HE4 and CA125 levels increase gradiently with rising risk, which can directly reflect tumor burden and surgical difficulty, highly consistent with previous studies (15, 16). Saffarieh et al. (15) reported that HE4 combined with CA125 showed favorable predictive performance for optimal cytoreduction, consistent with the dose-response trend in our study. Feng et al. (16) constructed a prediction model based on HE4 and CA125, also verifying that the combination could effectively distinguish patients with optimal and suboptimal cytoreduction, further supporting the reliability of our results. Mechanistically, CA125, a classic ovarian cancer marker, broadly reflects tumor burden, abdominal dissemination and lesion invasiveness, and is valuable for evaluating overall surgical difficulty and resection extent (17, 18). HE4 is specifically secreted by ovarian cancer cells and participates in key processes such as extracellular matrix degradation, peritoneal adhesion and invasive migration with higher specificity, compensating for the non-specific elevation of CA125 in benign diseases (19, 20). The combination of the two markers achieves complementary advantages, effectively improving the stability and accuracy of preoperative risk stratification and providing reliable reference for clinical judgment of tumor resectability.

Elevated platelet count was identified as an independent risk factor in this study, with each 10×10^9^/L increase in platelets associated with a 27% higher risk of suboptimal cytoreduction. Platelets promote extensive tumor implantation and metastasis through multiple pathways, including shielding tumor cells from NK cell immune surveillance, releasing vascular endothelial growth factor and platelet-derived growth factor to stimulate angiogenesis, and enhancing adhesion between tumor cells and peritoneal mesothelial cells (21, 22). Pankowska et al. (23) systematically illustrated the pro-invasive role of platelets in the ovarian cancer tumor microenvironment, indicating that elevated platelets are a peripheral blood marker of highly invasive and metastatic tumors. Muhammad et al. (24) also confirmed significantly higher preoperative HE4, CA125 and inflammation-related indicators in patients with suboptimal cytoreduction, consistent with our findings. Isingizwe et al. (25) further found enhanced platelet aggregation activity in ovarian cancer patients, exacerbating tumor invasion and dissemination. Incorporating platelets into the combined model increased the AUC from 0.79–0.88 for single markers to 0.95 with markedly improved specificity, suggesting that combining coagulation-inflammatory indicators with tumor markers more comprehensively and objectively reflects tumor biological behavior and enhances overall preoperative evaluation performance.

The triple-marker model constructed in this study shows preliminary promise and may eventually serve as a tool to assist treatment strategy selection and surgical planning in advanced ovarian cancer, but it currently lacks the external validation necessary to support direct clinical implementation. If externally validated, this model—requiring only routine preoperative serum testing—would be non-invasive, convenient, and potentially reproducible, and could eventually complement imaging by compensating for its insufficient sensitivity in detecting tiny implants and reducing subjective bias from physicians (26). In exploratory analyses, the model demonstrated high specificity that, if confirmed in external cohorts, could potentially enable stratification of high- and low-risk patients: hypothetically, low-risk patients might be candidates for primary cytoreductive surgery with standard resection extent, while high-risk patients could be considered for extended resection or neoadjuvant chemotherapy to reduce complication risks. These applications remain speculative pending prospective and external validation. To assess the rationality of the AUC in our study, we systematically compared our results with previous literature. Klotz et al. (27) confirmed that preoperative HE4 combined with CA125 achieved an AUC of 0.86 in predicting ovarian cancer surgical outcomes, similar to the performance of our combined model. Si et al. (28) constructed a nomogram for predicting ovarian cancer cytoreduction outcomes and also identified HE4 as a core independent risk factor, further validating the important role of HE4 in preoperative evaluation. Angioli et al. (29) reported an AUC of 0.86 for HE4 in predicting optimal cytoreduction in 57 patients with advanced ovarian cancer. Saffarieh et al. (15) found an AUC of 0.82 for HE4 in 110 patients. All these studies were single-center, small-sample retrospective analyses with AUC ranging from 0.71 to 0.86. The single-marker AUC of HE4 (0.79) and CA125 (0.88) in our study fell within the reasonable range reported previously. Notably, our study innovatively incorporated platelet count into the combined model, lifting the triple-marker AUC to 0.96. This improvement aligns with the trend reported by Muhammad et al. (24), who combined IL-6, CA125 and HE4 to predict tumor resectability in 64 patients and achieved an AUC as high as 0.989, suggesting that multi-marker combination strategies indeed significantly improve predictive performance. Lof et al. (30) validated the CONATS index (including HE4, age and WHO performance status) in 273 patients undergoing interval cytoreductive surgery with an AUC of 0.80, further confirming the clinical value of serum-based combined models and indicating that AUC may tend to be conservative in large-sample studies. The excellent performance of our combined model may be attributed to: (1) platelet count, as a peripheral marker of tumor microenvironment invasiveness, providing complementary biological information to HE4 and CA125; (2) strict inclusion and exclusion criteria ensuring homogeneous study population; (3) all surgeries performed by the same gynecologic oncology team with high standardization of surgical quality. However, it must be acknowledged that the absence of surgical complexity variables—such as the Peritoneal Cancer Index (PCI) or Aletti Surgical Complexity Score, which were not systematically collected in this retrospective cohort—means that our model captures the biological aggressiveness reflected by serum markers but not the anatomical dimension of disease distribution that strongly influences surgical difficulty. More importantly, this single-team surgical standardization, while a strength in minimizing inter-surgeon variability, may have inadvertently created a “closed-system” effect that artificially inflated the predictive performance of preoperative biomarkers, as surgical aggressiveness was held relatively constant. In other words, when surgical technique and aggressiveness are held relatively constant, preoperative biomarkers become the dominant determinants of residual disease—an artificial inflation of predictive performance that may not replicate in settings with greater surgical heterogeneity. Consequently, the excellent AUC of 0.959 observed in this internal validation should be viewed as hypothesis-generating and center-specific; rigorous external validation in independent multicenter cohorts with diverse surgical practices is essential before any claims of generalizability can be made.

This study has several limitations. First, this was a single-center retrospective study with a relatively small sample size (n=102), including only 37 events (suboptimal cytoreductions) and insufficient representation of non-serous pathological types (n=15). The limited sample size constrains the statistical power for detecting modest effect sizes and increases the risk of model overfitting, despite our use of bootstrap internal validation. More importantly, the single-center design inevitably introduces selection bias, as our patient population, surgical criteria, and perioperative management protocols reflect the practices of a single institution with a highly specialized gynecologic oncology team. This limits the direct generalizability of our findings to other centers with different patient demographics, surgical expertise, or treatment algorithms. Beyond the recognized risk of overfitting optimism given the event-per-variable ratio (37 events for up to 5 candidate predictors), the most critical caveat is that our model’s excellent AUC of 0.959, while internally consistent, has not been tested in an independent external cohort. Without such validation, this high estimate should be viewed as hypothesis-generating rather than a reflection of its true performance in unselected, real-world populations. The performance gap between internal validation and external application can be substantial, especially when case mix, surgical criteria, or laboratory assays differ across institutions. Therefore, while the model holds promise as a preoperative decision-support tool, its immediate clinical generalizability remains unproven, and our AUC should be interpreted with caution until validated in diverse, independent patient cohorts. Furthermore, no surgical complexity variables were included in this study, such as operative time, estimated blood loss, extent of peritoneal stripping, number of resected organs, or performance of upper abdominal procedures. These factors are known to be strongly associated with the likelihood of achieving optimal cytoreduction and represent an important dimension of surgical outcome prediction. Their omission means that our model captures only preoperative tumor burden and biological aggressiveness, but not the technical difficulty or anatomical challenges encountered during surgery. Future iterations of this model should incorporate surgical complexity scores or intraoperative assessment tools to provide a more comprehensive preoperative risk stratification framework. Long-term survival follow-up was not performed, so the predictive value of the model for progression-free survival and overall survival could not be determined. Confounding factors such as surgeon experience and perioperative management were not analyzed, which may affect the stability of the results. Furthermore, the generalizability of our findings to other centers and surgical teams requires external validation studies, which we have planned as the next step in our research program. We acknowledge that multicenter prospective studies with larger, more diverse patient populations are essential to confirm the external validity of our model before it can be considered for broader clinical adoption. Future research directions include: conducting multi-center prospective studies to expand sample size and include more pathological subtypes to verify external validity; combining imaging features and surgical complexity scores to build a multi-dimensional evaluation system for further improved predictive accuracy; developing a bedside rapid calculation tool for clinical popularization; incorporating long-term survival data to explore the extended value of the model from surgical guidance to prognosis prediction, providing more comprehensive evidence for individualized diagnosis and treatment of advanced ovarian cancer.

## 5 Conclusion

In this single-center retrospective analysis of 102 patients with advanced epithelial ovarian cancer, elevated preoperative serum HE4, CA125 levels and platelet count were identified as independent risk factors for suboptimal cytoreduction. HE4 and CA125 levels are linearly and positively correlated with the risk of suboptimal cytoreduction in a significant dose-dependent manner. The combined prediction model based on the three indicators achieved an AUC of 0.959 and a specificity of 95.4% in internal validation, with favorable calibration and net clinical benefit in this cohort. These findings are preliminary and require confirmation in independent external datasets before any claims of robustness can be made. If externally validated, a model requiring only routine preoperative serum testing could eventually provide a non-invasive, convenient, and reproducible quantitative reference to assist in preoperative risk assessment. At present, however, it should be regarded as an exploratory tool rather than a definitive guide for clinical decision-making between primary cytoreductive surgery and neoadjuvant chemotherapy. Whether this model can actually help improve the rate of optimal cytoreduction, reduce perioperative complications, or improve patient prognosis remains to be determined through prospective and externally validated studies.

## Data Availability

The raw data supporting the conclusions of this article will be made available by the authors, without undue reservation.
